# Poisonous Visiting Cards

**Published:** 1854-07

**Authors:** 


### POISONOUS VISITING CARDS.

Few ladies remember that they carry around poison in their card cases. But it is so, and sometimes to the danger of children or thoughtless people of larger growth. The elegant and highly polished enamel on visiting cards is composed, in part, of poisonous mineral substances, and if eaten would produce serious sickness. The manufacture of this card paper is said to be exceedingly unhealthy, and we may well believe it. It would be, therefore, a kind thing to the workmen engaged in the manufacture of cards, and a safe thing for themselves and their children, if the ladies, who set the fashion to these things, would give up the use of enameled cards, and confine themselves to those of plain surface. These, we understand, are now decidedly the most fashionable, from what cause we know not, but the plain, brownish cards are considered the most stylish. It is gratifying to see fashions turned in the channels of common sense, of health and humanity, even though in a small matter. We hope that the knowledge of the dangerous character of these cards will not lead to their restoration to the feminine favor and to fashion, which is a very fickle thing; we mean, of course, the fashion, not the fair.—*Providence Journal.*

